# miR-190 enhances endocrine therapy sensitivity by regulating SOX9 expression in breast cancer

**DOI:** 10.1186/s13046-019-1039-9

**Published:** 2019-01-18

**Authors:** Yue Yu, Wen Yin, Zhi-Hao Yu, Yan-Jun Zhou, Jiang-Rui Chi, Jie Ge, Xu-Chen Cao

**Affiliations:** 1 The First Department of Breast Cancer, Tianjin Medical University Cancer Institute and Hospital, National Clinical Research Center for Cancer, Huan-Hu-Xi Road, Hexi District, Tianjin, 300060 China; 2 Key Laboratory of Cancer Prevention and Therapy, Tianjin, 300060 China; 3 Tianjin’s Clinical Research Center for Cancer, Tianjin, 300060 China; 4 Key Laboratory of Breast Cancer Prevention and Therapy, Tianjin Medical University, Ministry of Education, Tianjin, 300060 China

**Keywords:** Breast cancer, Endocrine therapy, Wnt/β-catenin signaling, miR-190, SOX9, ZEB1

## Abstract

**Background:**

Breast cancer is the most common cancer among women worldwide, and approximately 70% of breast cancers are hormone receptor-positive and express estrogen receptor-α (ERα) or/and progesterone receptor. Therapies targeting ERα have been successfully used in patients with ERα^+^ breast cancer. However, intrinsic or acquired resistance to anti-estrogen therapy presents a major challenge. The Wnt/β-catenin signaling pathway regulates various processes that are important for cancer progression, and emerging evidences have shown a close interaction between Wnt/β-catenin and ERα signaling. miR-190 is also involved in ER signaling and our previous study indicated that miR-190 suppresses breast cancer metastasis.

**Methods:**

The effect of miR-190 on breast cancer anti-estrogen sensitivity was investigated both in vitro and in vivo. The protein expression levels and localization were analyzed by western blotting and immunofluorescence, respectively. Chromatin immunoprecipitation and dual-luciferase reporter assays were used to validate the regulation of the zinc-finger E-box binding homeobox 1/ ERα-miR-190-SRY-related high mobility group box 9 (ZEB1/ERα-miR-190-SOX9) axis.

**Results:**

miR-190 increased the anti-estrogen sensitivity of breast cancer cells both in vitro and in vivo. miR-190 inhibited Wnt/β-catenin signaling by targeting SOX9, and its expression inversely correlated with that of SOX9 in breast cancer samples. Furthermore, ERα and ZEB1 competitively regulated miR-190 expression.

**Conclusions:**

Our data uncover the ZEB1/ERα-miR-190-SOX9 axis and suggest a mechanism by which the Wnt/β-catenin signaling pathway is involved in breast cancer anti-estrogen therapy.

**Electronic supplementary material:**

The online version of this article (10.1186/s13046-019-1039-9) contains supplementary material, which is available to authorized users.

## Introduction

Breast cancer is the most frequently diagnosed malignancy in women worldwide [1]. It is the most common malignant tumor, and the third largest cause of cancer-related deaths in China. Although the incidence of this disease is increasing, the number of deaths caused by it is decreasing [2]. Approximately 70% of breast cancers are hormone receptor-positive and express estrogen receptor-α (ERα) or/and progesterone receptor. ERα is a nuclear receptor and is a key regulator of breast cancer development and progression. Therapies targeting ERα have been successfully applied in patients with ERα^+^ breast cancer [3]. However, intrinsic or acquired resistance to anti-estrogen therapy presents a major challenge. Thus, an improved understanding of the ERα-related regulation network may reveal new strategies for breast cancer endocrine therapy.

miRNAs are a class of small, endogenous, non-coding RNAs that negatively regulate the expression of a wide variety of genes by binding to complementary sequences in the 3′-untranslated regions (UTRs) of target mRNAs [4, 5]. A large number of studies have shown that miRNA alteration or dysfunction is involved in cancer development and progression by regulating cancer cell proliferation, differentiation, apoptosis, angiogenesis, metastasis, and metabolism [6, 7]. Dysregulated miRNAs are involved in breast cancer carcinogenesis and progression and function as oncogenes or tumor suppressors, as well as useful biomarkers in the diagnosis and prognosis of breast cancer [8, 9]. miR-190 is located in the intron region of the talin2 (TLN2) gene on chromosome 15q22.2. Previous studies have shown that the expression of miR-190 is reduced in aggressive neuroblastomas, and its overexpression leads to repression of tumor growth and prolonged dormancy periods in fast-growing tumors [10]. miR-190 suppresses the migration, invasion, and angiogenesis abilities of hepatocellular carcinoma cells through inhibition of epithelial–mesenchymal transition (EMT) phenotype [11]. In contrast, miR-190 expression is elevated in gastric cancer tissues and contributes to gastric cancer progression [12], suggesting that miR-190 may play a different role in different stages of tumor development and different tumor environments. Our previous study indicated that miR-190 suppresses breast cancer metastasis by regulation of transforming growth factor-β (TGF-β)-induced EMT [13]. The expression of circulating miR-190 is lower in breast cancer patients with early relapse compared to those without early relapse [14]. miR-190 is also involved in ER signaling, causing inhibition of breast cancer metastasis [15]. Thus, we speculated that miR-190 is involved in the ERα-related regulation network in breast cancer.

In this study, we investigated the effect of miR-190 on endocrine therapy resistance in breast cancer. miR-190 decreases the stemness and the activation of Wnt signaling, resulting in enhancement of endocrine therapy sensitivity by targeting SRY-related high mobility group box 9 (SOX9). We further demonstrated a mechanism for zinc-finger E-box binding homeobox 1 (ZEB1)-miR-190-SOX9 axis-mediated resistance to endocrine therapy in breast cancer. ZEB1 binds to the miR-190 promoter region, competitively inhibiting ERα binding, and resulting in resistance to endocrine therapy. Therefore, our study revealed a novel mechanism of Wnt signaling pathway-mediated resistance to endocrine therapy in breast cancer.

## Materials and methods

### Antibodies, reagents, plasmids, miRNA, and small interfering RNA (siRNA)

The antibodies, reagents, plasmids, miRNAs, and siRNAs used in this study are listed in the Additional file 1: Supplementary Materials and Methods.

### Cell culture and clinical samples

The human breast cancer cell lines MCF7, T47D, MDA-MB-453, MDA-MB-468, MDA-MB-231, and MDA-MB-435 were obtained from the Cell Bank of the Chinese Academy of Sciences (Shanghai, China). Culture conditions have been described in the Additional file 1: Supplementary Materials and Methods.

Breast cancer specimens were obtained from Tianjin Medical University Cancer Institute and Hospital (TMUCIH). Thirty primary breast cancer tissue samples were used for this study. All tumor samples were obtained from patients newly diagnosed with breast cancer and who had received no therapy before sample collection. This study was approved by the Institutional Review Board of TMUCIH and written consent was obtained from all participants.

### Transient and stable transfection of breast cancer cells

For transient transfection, miRNAs or siRNAs were transfected into different cell lines using FuGENE HD Transfection Reagent (Promega, Madison, WI, USA) and plasmids were transfected using TransFast Transfection Reagent (Promega) according to the manufacturer’s recommendations. To generate stable cells, lentiviruses (RiboBio, Shanghai, China) were used to infect MDA-MB-231 cells according the manufacturer’s recommendations.

### Cell proliferation assay

MTT, plate colony formation, and EdU assays were performed to evaluate cell proliferation, as previously described [13]. Experiments were carried our as described in the Additional file 1: Supplementary Materials and Methods.

### Mammosphere forming assay

Single cells were plated at 10, 000 cells/ml on 6-well plate in serum-free DMEM/F12 supplemented with 20 ng/ml EGF, 4 mg/ml insulin, 5 μg/ml heparin (Sigma-Aldrich), 1 μg/ml hydrocortisone, 0.5% BSA (Sigma-Aldrich) and B27 (Sigma-Aldrich). Fresh medium was supplemented every three days. The mammosphere formation efficiency (shown as percentage) was calculated by dividing the number of mammospheres formed by the original number of single cells seeded.

### Western blotting and immunofluorescence

Cells were lysed in protein lysis buffer [20 mM Tris-HCl (pH 7.4), 5 mM EDTA, 1% Triton X-100, 150 mM NaCl, and 1% DTT] containing a protease inhibitor cocktail tablet (Roche Molecular Biochemicals, Indianapolis, IN, USA). Proteins were resolved by sodium dodecyl sulfate-polyacrylamide gel electrophoresis, transferred onto polyvinylidene fluoride membranes (Millipore, Bedford, MA, USA), and incubated with primary antibodies overnight at 4 °C, followed by incubation with horseradish peroxidase-conjugated secondary antibody. The blots were visualized with ECL reagent (Millipore).

For immunofluorescence analysis, cells were seeded onto glass coverslips in 24-well plates, washed with phosphate buffered saline (PBS), fixed in 4% formaldehyde solution for 30 min, and then permeabilized with 0.2% Triton X-100/PBS for 15 min. Cells were blocked with 2% bovine serum albumin in PBS for 30 min. Coverslips were incubated with primary antibodies overnight at 4 °C, followed by incubation with FITC-/TRITC-conjugated secondary antibodies for 1 h at room temperature and then stained with 4′,6-diamidino-2-phenylindole. Finally, coverslips were observed under a fluorescence microscope.

### RNA extraction and reverse transcription quantitative polymerase chain reaction (RT-qPCR)

Total RNA of cultured cells, surgically resected fresh breast tissues, and formalin-fixed paraffin-embedded clinical specimens were extracted using mirVana PARIS kit (Life Technologies) according to the manufacturer’s recommendations. qPCR was performed to detect mRNA expression using GoTaq qPCR Master Mix (Promega). TaqMan RT-qPCR was performed to detect mature miRNA expression using TaqMan miRNA reverse transcription kit, has-RNU6B (U6, ABI Assay ID: 001093), and miR-190 (ABI Assay ID: 000489) according to the manufacturer’s protocol (Life Technologies). The sequences of PCR primers are listed in Additional file 1: Table S1.

### Chromatin immunoprecipitation (ChIP) analysis

ChIP assay was performed according to the protocol of Upstate Biotechnology, as previously described [16]. The primer sequences used for miR-190 promoter were 5′-GAATGGCTCATGGTCTTTG-3′ and 5′-GCAGCAACTCCGATAACTG-3′ (ZEB1), 5′-GACAGTTATCGGAGTTGCT-3′ and 5′-CGTGTTCTTTCCTGTTGCC-3′ (ERα).

### Luciferase reporter assays

Luciferase assays were carried out using a dual luciferase assay kit according to the manufacturer’s recommendations, as previously described [17].

### Xenograft

Stable miR-190-overexpressing MDA-MB-231 and control cells (1 × 10^6^ cells) together with 100 μg of Matrigel (BD Biosciences, San Diego, CA, USA) were inoculated into the mammary fat pads of 5-week-old female BALB/c mice. Tumor development was allowed to reach a volume of ~ 100 mm^3^. The mice were then randomized into 2 groups, and placebo or tamoxifen pellets (5 mg/pellet) were subcutaneously embedded for another 3 weeks. Tumor growth was recorded once a week with a caliper-like instrument. Tumor volume was calculated according to the formula volume = (width^2^ × length)/2. Eight weeks after inoculation, mice were killed, and the final volume and weight of tumor tissues were determined. All animal experimental protocols were approved by the Animal Ethics Committee of TMUCIH.

### Statistical analysis

Data are presented as mean ± standard deviation. The student’s *t*-test (2-tailed) was used to determine differences between the experimental and control groups. The level of significance was set to *P* < 0.05. Spearman’s correlation was used to test the significance of association between miR-190 and SOX9 expression. All calculations were performed with the SPSS for Windows statistical software package (SPSS Inc., Chicago, IL, USA).

## Results

### miR-190 increases the anti-estrogen sensitivity of breast cancer cells in vitro

**Fig. 1:**
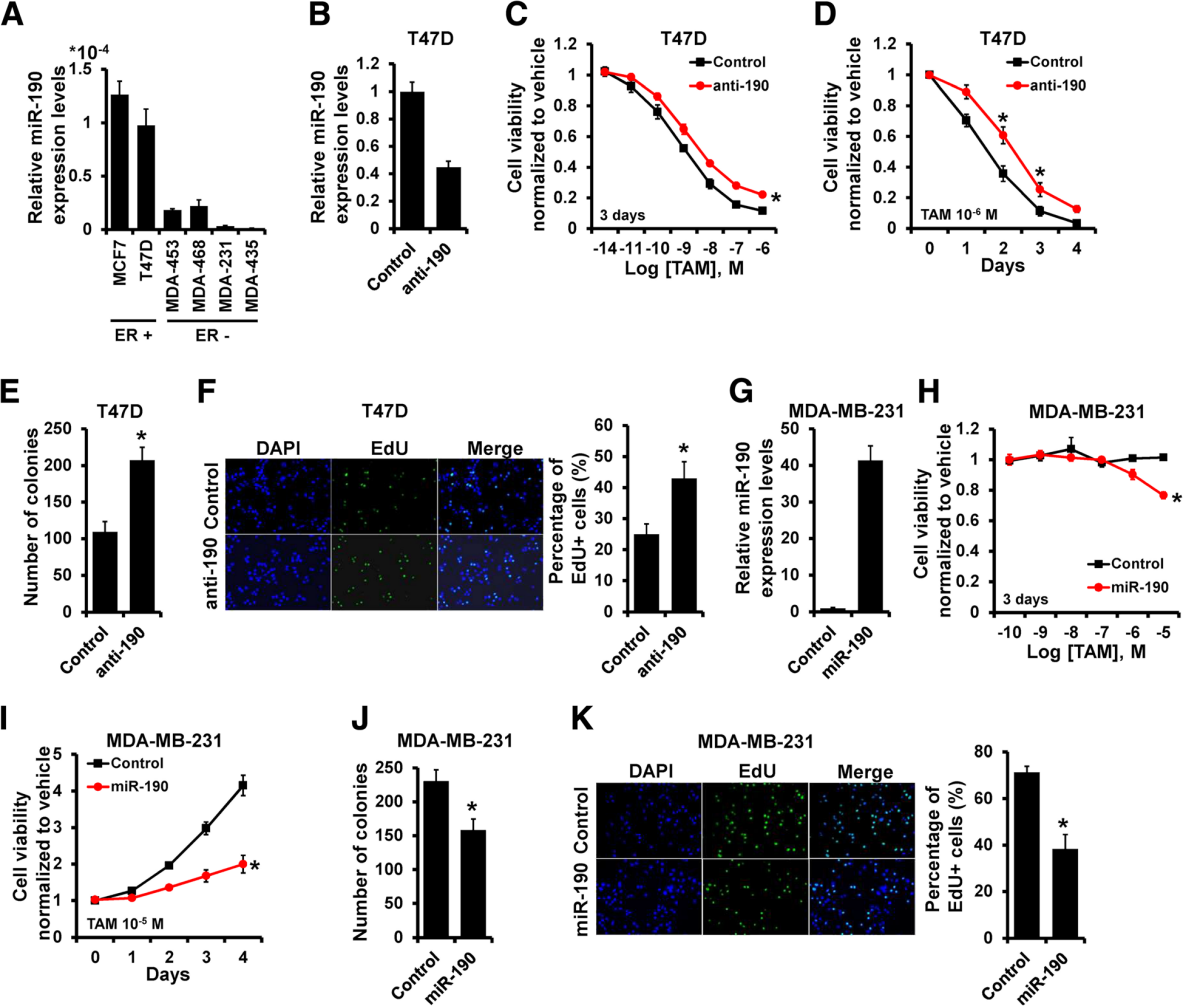
miR-190 increases tamoxifen sensitivity of breast cancer cells in vitro. **a**, miR-190 expression in breast cancer cell lines as determined by RT-qPCR. **b**, miR-190 expression in T47D cells transfected with miR-190 inhibitor as determined by RT-qPCR. **c–f**, Cell growth inhibition was determined by MTT (**c** and **d**), colony formation (**e**), and EdU (**f**) assays in T47D cells transfected with miR-190 inhibitor, as well as in control cells after treatment with tamoxifen. **G**, miR-190 expression in MDA-MB-231 cells transfected with miR-190 mimics as determined by RT-qPCR. **h–k**, Cell growth inhibition was determined by MTT (**h** and **i**), colony formation (**j**), and EdU (**k**) assays in MDA-MB-231 cells transfected with miR-190 mimics, as well as in control cells after treatment with tamoxifen. *P < 0.05

Our previous study indicated that miR-190 did not alter the breast cancer proliferation [13], however, we observed that the expression of miR-190 was down-regulated in all ER- breast cancer cell lines (MDA-MB-453, MDA-MB-468, MDA-MB-231 and MDA-MB-435) as compared to ER+ breast cancer cell lines (MCF7 and T47D; Fig. 1a), suggesting that miR-190 may influence the breast cancer endocrine therapy sensitivity. Next, we investigated the influence of miR-190 on endocrine therapy sensitivity by transfecting T47D cells with miR-190 inhibitor (Fig. 1b). The miR-190-depleted T47D and control cells were treated with tamoxifen and cell viability was measured. miR-190 depletion rendered T47D cells less sensitive to tamoxifen (Fig. 1c and d). The colony assay also indicated that the number of colonies was higher in miR-190-depleted T47D cells compared to that in control cells (Fig. 1e). EdU assay further revealed that depletion of miR-190 markedly increased the number of cells in the S phase after treatment with tamoxifen (Fig. 1f). Conversely, miR-190 overexpression in MDA-MB-231 cells (Fig. 1g) decreased cell viability (Fig. 1h and i), the number of colonies (Fig. 1j), and the percentage of cells in the S phase (Fig. 1k). The similar phenomena was also observed in MCF7 and MDA-MB-435 cells (Additional file 1: Figure S1). Together, these results suggest that miR-190 enhances endocrine therapy sensitivity in breast cancer cells in vitro.

### Overexpression of miR-190 increases the anti-estrogen sensitivity of breast cancer cells in vivo

**Fig. 2:**
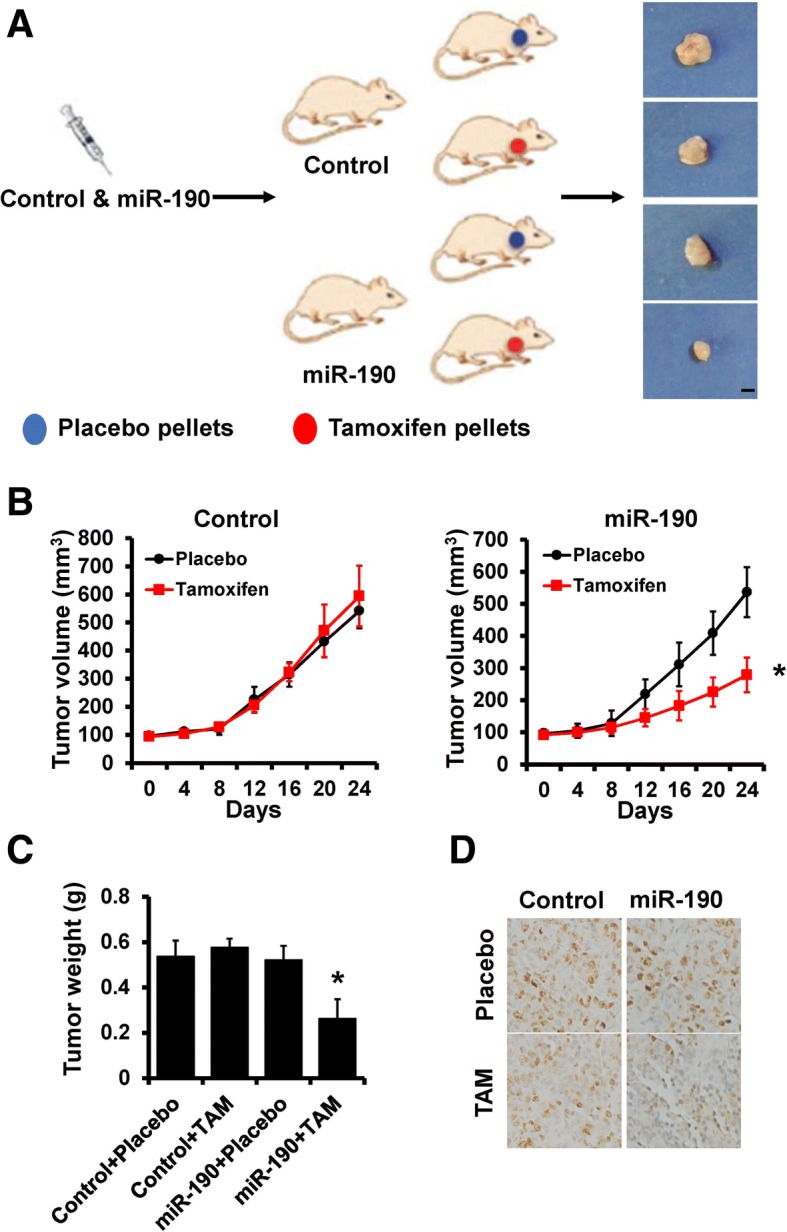
Overexpression of miR-190 increases tamoxifen sensitivity in vivo. **a**, A total of 1 × 10^6^ 231-miR-190 or control cells were injected into the mammary fat pads of nude mice. When the tumor volume was ~ 100 mm^3^, the mice were divided into two groups and treated with tamoxifen and placebo, respectively. Representative photos of the tumors formed by 231-miR-190 or control cells at harvest time. **b**, Tumor volume of xenograft mice injected with 231-miR-190 (right) or control (left) cells treated with tamoxifen or placebo at the indicated times. **c**, The weights of tumors formed by 231- miR-190 or control cells treated with tamoxifen or placebo at harvest time. **d**, The expression of Ki-67 in tamoxifen- or placebo-treated xenograft tumors was examined by immunohistochemical staining. *P < 0.05

Next, we assessed whether miR-190 overexpression in breast cancer cells can influence tumor response to anti-estrogen treatment in vivo. To achieve this, 231-miR-190 or 231-control cells were injected into the mammary fat pads of female BALB/c nude mice to establish tumors, and mice were subsequently treated with tamoxifen (Fig. 2a). As shown in Fig. 2b and c, tumor volumes and weights were significantly decreased in mice injected with 231-miR-190 cells compared with those in mice injected with 231-control cells after treatment with tamoxifen. The expression of Ki-67 was also decreased in tumors from 231-miR-190 mice compared to that in tumors from 231-control mice, as evidenced by immunohistochemical staining (Fig. 2d). Collectively, these results indicate that overexpression of miR-190 enhances endocrine therapy sensitivity in breast cancer cells in vivo.

### SOX9 is a direct target of miR-190

**Fig. 3:**
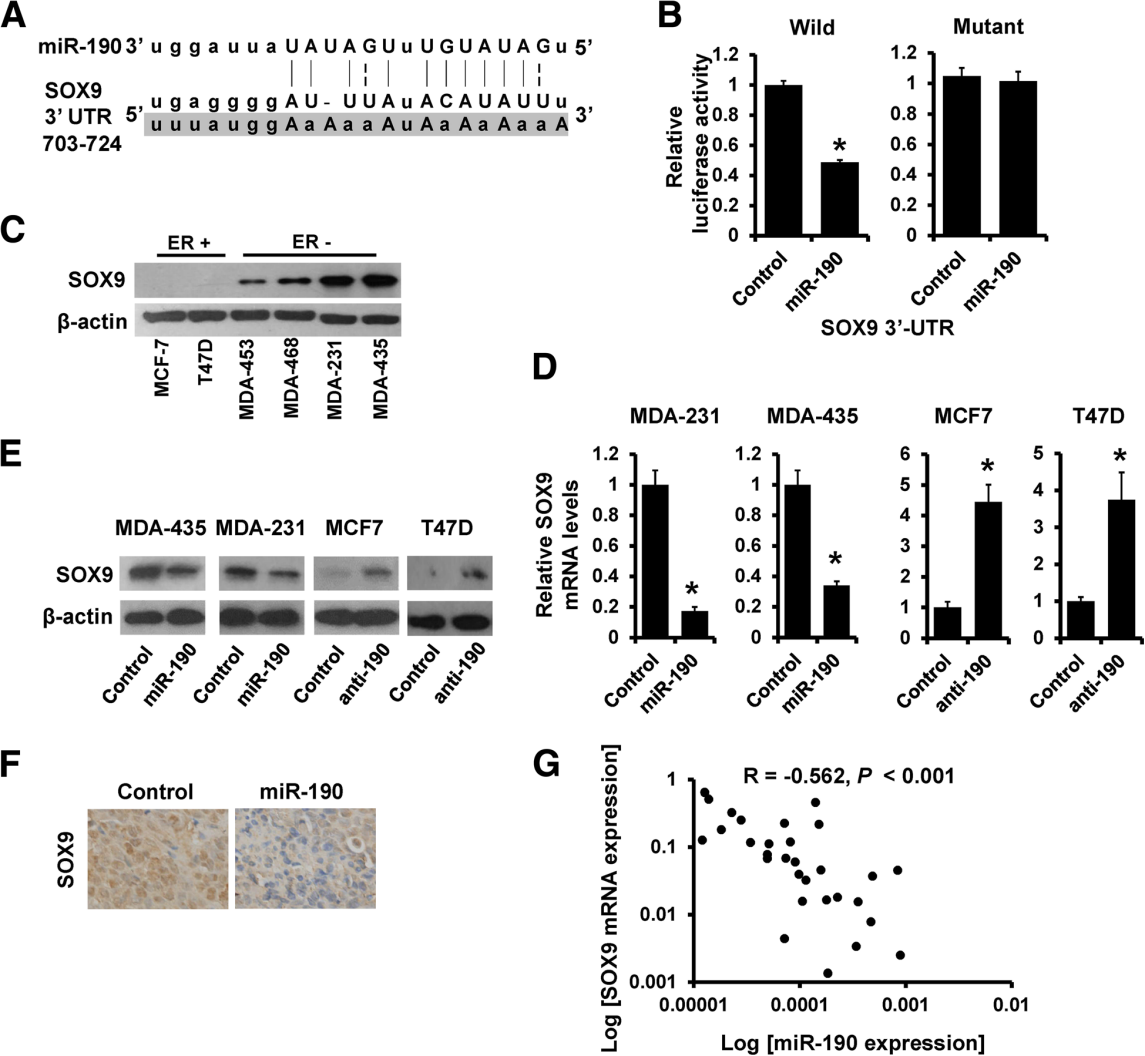
SOX9 is a direct target of miR-190. **a**, The predicted binding of miR-190 with SOX9 3′-UTR. **b**, Dual luciferase reporter assay was performed to validate the miR-190 target, SOX9. **c**, The expression of SOX9 in breast cancer cell lines as determined by western blotting. **d**, miR-190 expression in indicated cells as determined by RT-qPCR. **e**, The expression of SOX9 in indicated cells as determined by western blotting. **f**, The expression of SOX9 in 231-miR-190 and 231-control xenograft tumors was examined by immunohistochemical staining. **g**, The relationship between miR-190 and SOX9 mRNA expression as determined by RT-qPCR. *P < 0.05

To elucidate the biological mechanisms underlying the role of miR-190 in anti-estrogen sensitivity of breast cancer cells, we investigated the potential targets of miR-190. The target prediction program, TargetScan, was applied to identify SOX9 as a putative miR-190 target. To further confirm this regulation, SOX9 3′-UTR and its mutant containing the putative miR-190 binding sties were cloned into the downstream luciferase ORF (Fig. 3a). As compared to that in control cells, the luciferase activity was significantly decreased in miR-190-transfected 293FT cells with inhibition rates of 50% (Fig. 3b, left). This effect was abolished in mutated SOX9 3′-UTR, in which the binding site for miR-190 was inactivated by site-directed mutagenesis (Fig. 3b, right). Contrary to the expression of miR-190 as determined by RT-qPCR (Fig. 1a), western blot analysis revealed that the expression of SOX9 was much higher in ER^−^ breast cancer cell lines compared with that in the ER^+^ breast cancer cell lines (Fig. 3c). Furthermore, the expression of SOX9 was decreased in miR-190-overexpressing MDA-MB-231 and MDA-MB-435 cells and was increased in miR-190-depleted MCF7 and T47D cells compared with that in control cells, as determined by RT-qPCR (Fig. 3d) and western blotting (Fig. 3e). Immunohistochemical staining confirmed the down-regulation of SOX9 in tumors from 231-miR-190 mice compared to tumors from 231-control mice (Fig. 3f). To further investigate the clinical relationship between miR-190 and SOX9, we examined the expression of SOX9 and miR-190 in 30 specimens of primary breast cancer tissues by RT-qPCR. As shown in Fig. 3g, SOX9 expression exhibited a significant negative relationship with miR-190 expression. Together, these results indicated that SOX9 is a direct target of miR-190.

### miR-190 affects anti-estrogen sensitivity and stem cell content by suppressing SOX9

**Fig. 4:**
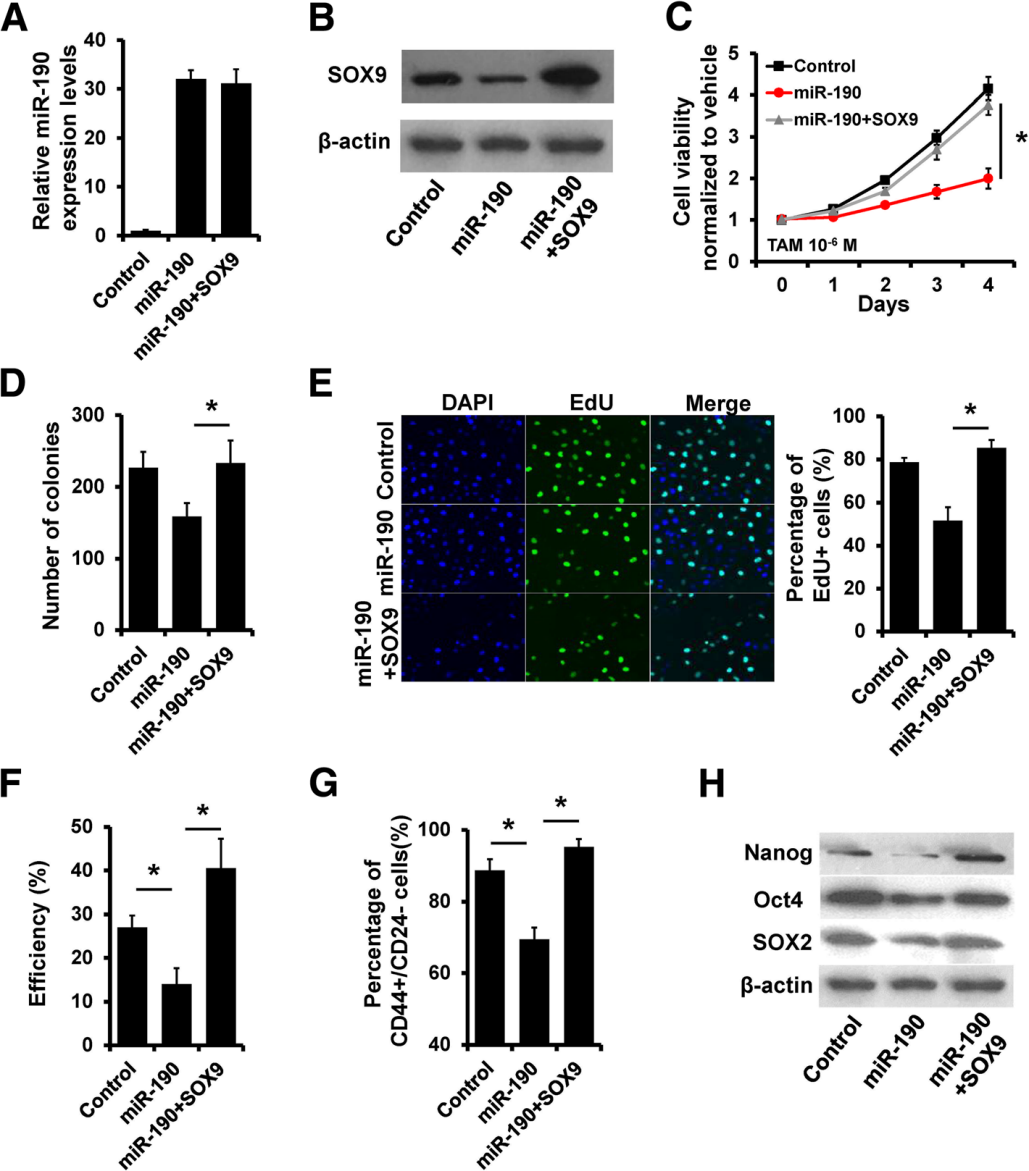
SOX9 rescues the effect of miR-190 on tamoxifen sensitivity and stemness. **a**, miR-190 expression in indicated MDA-MB-231 cells as determined by RT-qPCR. **b**, SOX9 expression in indicated MDA-MB-231 cells as determined by western blotting. **c~e**, Cell growth inhibition was determined by MTT (**c**), colony formation (**d**), and EdU **(e**) assays in MDA-MB-231 cells transfected with miR-190 mimics and SOX9 plasmid, as well as in control cells after treatment with tamoxifen. **F**, Mammosphere formation assay of cells as in (**a**). **g**, CD44^high^/CD24^low^ CSC population analysis of cells as in (**a**). **h**, The expression of CSC markers in cells as in (**a**) was determined by western blotting. *P < 0.05

To corroborate that SOX9 mediates the role of miR-190 in anti-estrogen responses in breast cancer cells, we transfected SOX9 mammalian plasmid into MDA-MB-231 cells in addition to miR-190 overexpression. While miR-190 overexpression led to SOX9 repression, transient transfection of SOX9 plasmid restored the expression of SOX9 (Fig. 4a and b). SOX9 overexpression abolished the effects of miR-190 on tamoxifen sensitivity in MDA-MB-231 cells (Fig. 4c–e). Breast cancer stem cells have been reported to lack ER or express it at very low levels, which may facilitate the resistance of cancer stem cells to the anti-proliferative effects of tamoxifen [18]. We next determined the relevance of miR-190-SOX9 regulation in the acquisition of a cancer stem cell phenotype. Overexpression of miR-190 led to a significant inhibition of mammosphere formation (Fig. 4f) and a significant reduction in the percentage of CD44^high^/CD24^low^ cancer stem cell (CSC) population (Fig. 4g). With forced expression of SOX9, miR-190 was no longer able to inhibit mammosphere formation and decrease the percentage of CSC population (Fig. 4f and g). The expression of CSC markers Nanog, Oct4, and SOX2 was also decreased in miR-190-overexpressing MDA-MB-231 cells compared to that in control cells, whereas forced SOX9 expression could rescue the expression (Fig. 4h). Conversely, knockdown of SOX9 eliminates the effect of miR-190 depletion on tamoxifen sensitivity and stemness in T47D cells (Additional file 1: Figure S2). Thus, these results indicated that miR-190 enhances endocrine therapy sensitivity and decreases stemness of breast cancer cells by suppressing SOX9.

### miR-190 inhibits Wnt/β-catenin signaling by down-regulation of SOX9

**Fig. 5:**
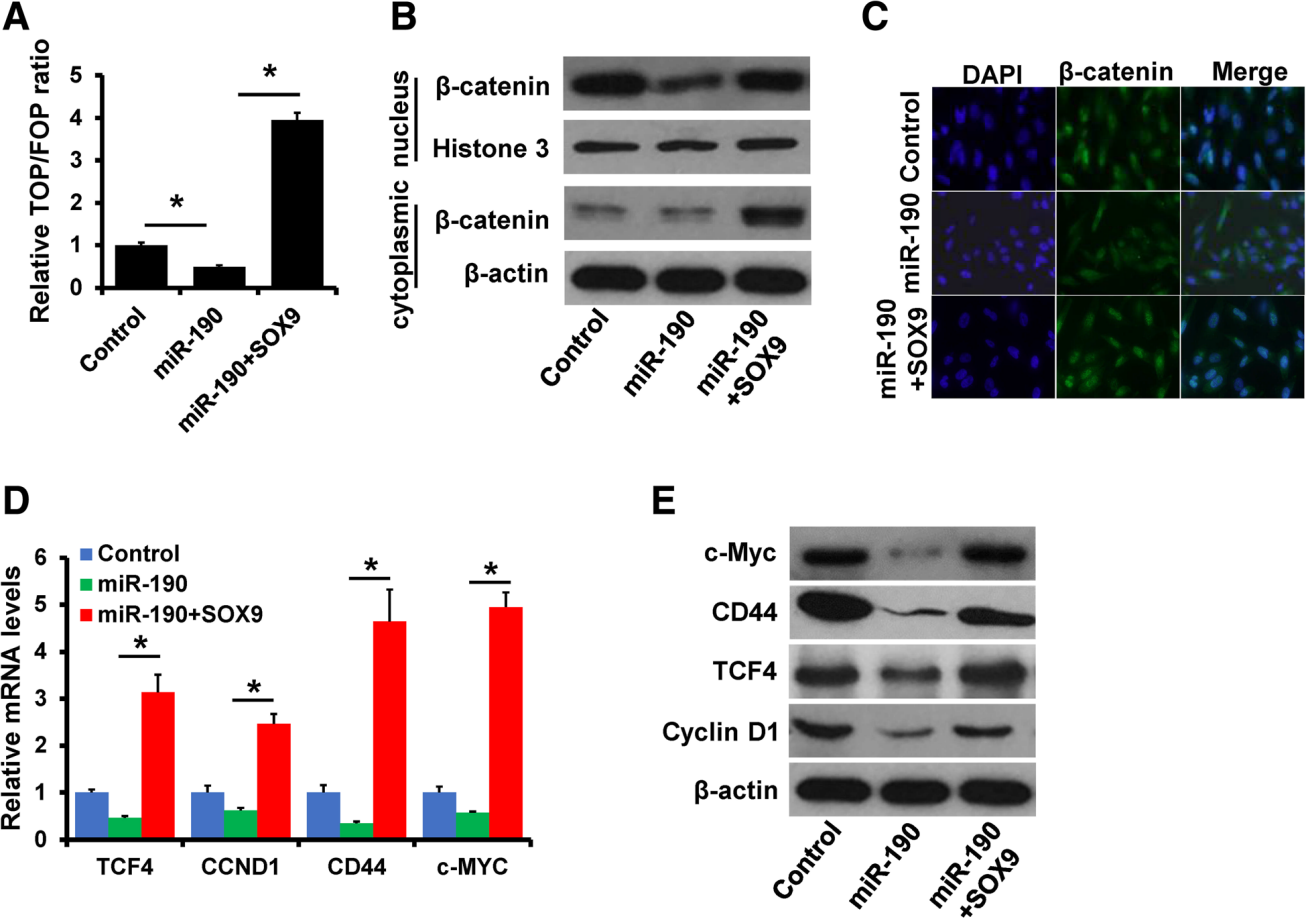
miR-190 inhibits Wnt/β-catenin signaling by regulating of SOX9. **a**, Luciferase reporter analysis of Wnt/β-catenin signaling in MDA-MB-231 cells transfected with miR-190 mimics and SOX9 plasmid, as well as in control cells. **b**, The expression of β-catenin in cells as in (**a**) was determined by western blotting. **C**, Localization of β-catenin in cells as in (**a**) was determined by immunofluorescence staining. **d** and **e**, The mRNA and protein expression of downstream targets of Wnt/β-catenin signaling in cells as in (**a**) was determined by RT-qPCR (**d**) and western blotting (**e**). *P < 0.05

SOX9 has been identified to drive Wnt/β-catenin signaling pathway activation and cancer progression [19, 20]. Thus, we next determined whether miR-190 regulates Wnt/β-catenin signaling by targeting SOX9. We detected the luciferase activity of TOP/FOP reporter in miR-190-overexpressing MDA-MB-231 cells with or without SOX9 overexpression. The luciferase activity was significantly decreased in miR-190-overexpressing MDA-MB-231 cells, whereas this effect was reversed following SOX9 overexpression (Fig. 5a). β-catenin has a dynamic signaling function in the Wnt/β-catenin pathway. Its elevated concentration and translocation to the nucleus are equivalent to the activated state of the pathway [21]. We investigated the effect of miR-190-SOX9 regulation on β-catenin nuclear translocation. Analysis of nuclear and cytoplasmic extracts by western blotting showed that overexpression of miR-190 markedly inhibited its nuclear translocation, whereas SOX9 expression induced its nuclear translocation (Fig. 5b). In addition, the nuclear localization of β-catenin was confirmed by immunofluorescence (Fig. 5c). We further determined the expression of Wnt/β-catenin target genes using RT-qPCR and western blotting. Overexpression of miR-190 decreased the mRNA and protein levels of c-Myc, CD44, TCF4, and cyclin D1 in MDA-MB-231 cells, whereas overexpression of SOX9 rescued the expression of these genes in miR-190-overexpressing MDA-MB-231 cells (Fig. 5d and e). Together, these results indicated that miR-190 inhibits Wnt/β-catenin signaling by targeting SOX9.

### ERα and ZEB1 competitively bind to the miR-190 promoter and regulate its expression

**Fig. 6:**
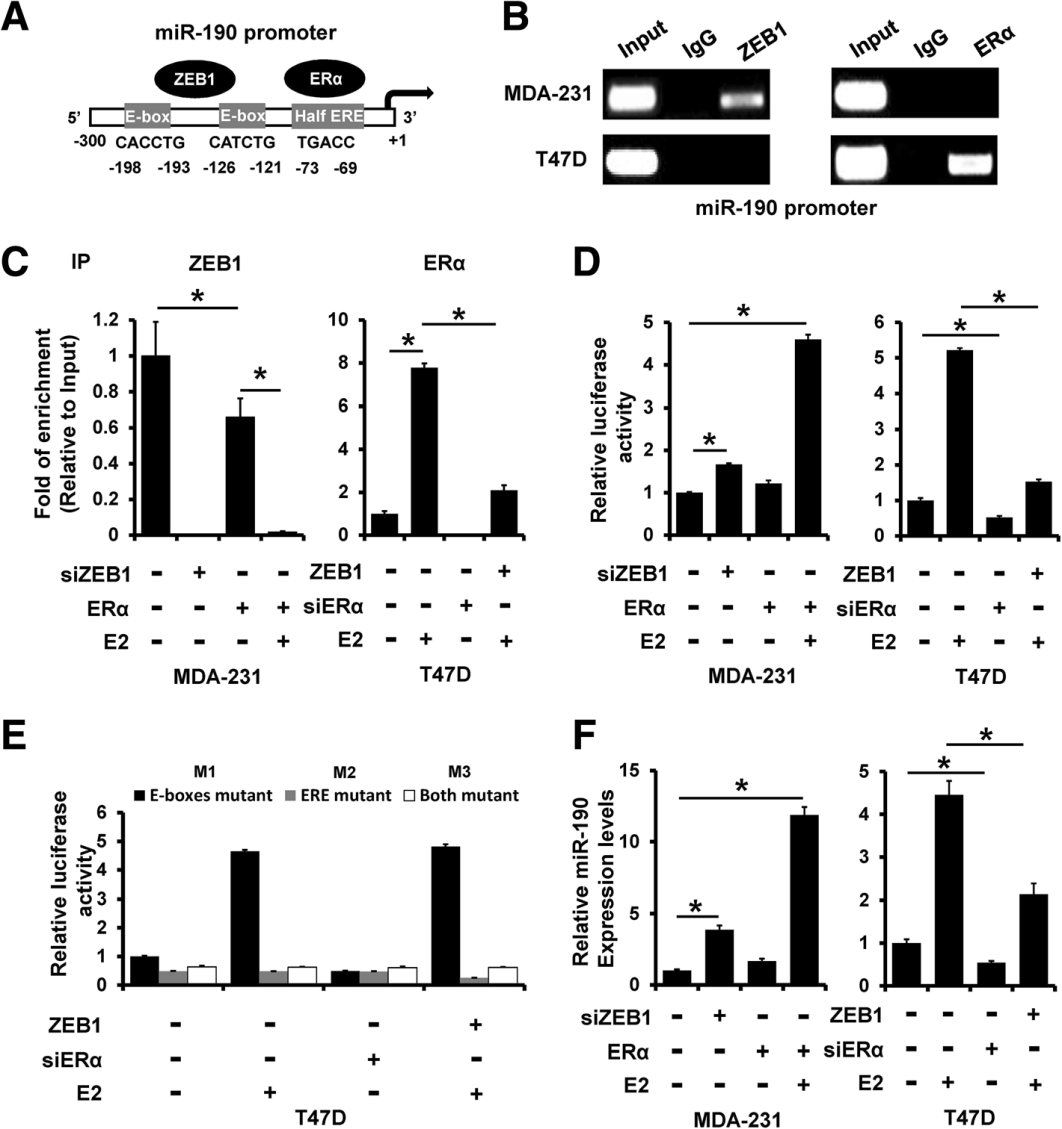
ERα and ZEB1 competitively bind to the miR-190 promoter and regulate its expression. **a**, Promoter analysis of miR-190. Two E-boxes and a half ERE were located on the core promoter region of miR-190 (− 300 to + 1). **b**, Interaction between ERα or ZEB1 and the miR-190 promoter sequence in MDA-MB-231 and T47D cells as determined by ChIP assay. **c**, Interaction between ZEB1 or ERα and the miR-190 promoter sequence in ERα-transfected MDA-MB-231 cells (left), ZEB1-transfected T47D cells (right), as well as in control cells as determined by ChIP assay. **d**, miR-190 promoter activity was measured in cells as in (**c**) by luciferase analysis. **e**, Mutation of E-box or/and ERE sequence prevents ZEB1 or ERα from regulating the miR-190 promoter. **f**, miR-190 expression in cells as in (**c**) was determined by RT-qPCR. *P < 0.05

We next examined the promoter sequence of miR-190 and, interestingly, found two E-boxes and a half estrogen response element (ERE) on the miR-190 promoter region (Fig. 6a). ChIP assay revealed that ZEB1 could bind to the miR-190 promoter in MDA-MB-231 cells, whereas ERα could bind to the miR-190 promoter in T47D cells (Fig. 6b). The binding of ZEB1 to the miR-190 promoter was decreased after transfection with ERα, and it was further decreased after treatment with E2 in MDA-MB-231 cells (Fig. 6c, left). Furthermore, overexpression of ZEB1 decreased the binding of ERα to the miR-190 promoter (Fig. 6c, right). To examine whether ZEB1 and ERα competitively regulate miR-190 promoter activity, we transfected siRNAs targeting ZEB1 or ERα plasmid into MDA-MB-231 cells or siRNAs targeting ERα or ZEB1 plasmid into T47D cells with or without E2 treatment. As shown in Fig. 6d, the luciferase activity was increased in ERα-transfected MDA-MB-231 cells (Fig. 6d, left), whereas it was decreased in ZEB1-transfected T47D cells (Fig. 6d, right). We next constructed three mutant clones in which the E-box sequence was mutated from CACCTG to TTTTTT (M1), ERE sequence was mutated from TGACC to TTTTT (M2), or which had both mutated clones (M3), and used them to transfect T47D cells. As shown in Fig. 6e, M3 mutant showed no altered luciferase expression after transfection with ZEB1 plasmid or siRNAs targeting ERα with or without E2 treatment. The expression of miR-190 was also affected by transfection with ZEB1, ERα, or siRNAs targeting ZEB1 or ERα in MDA-MB-231 or T47D cells (Fig. 6f). Together, these results indicated that ERα and ZEB1 competitively regulate miR-190 expression.

### miR-190 mediated the regulation of SOX9 expression via ERα and ZEB1

**Fig. 7:**
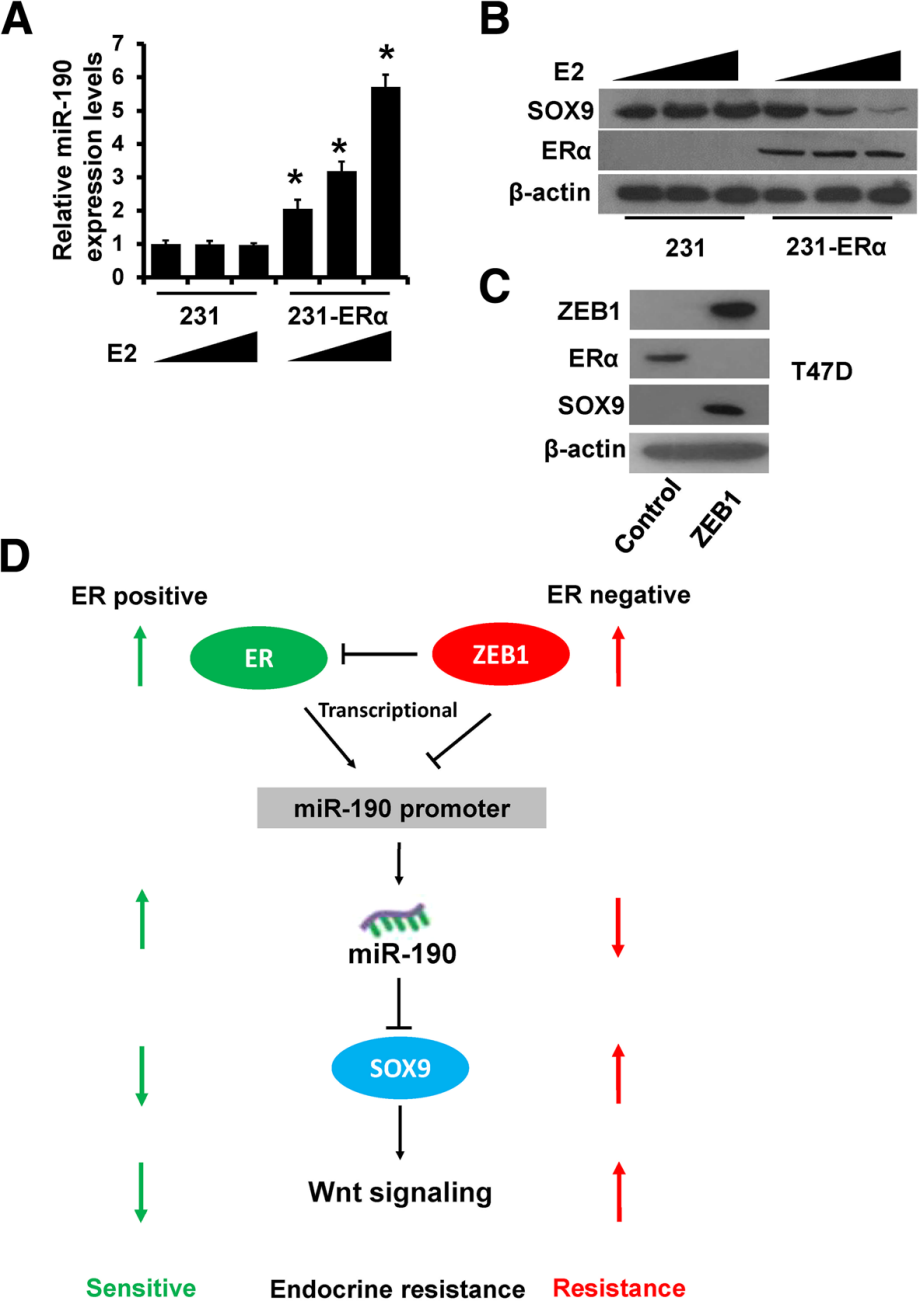
ERα and ZEB1 regulate SOX9 expression through miR-190. **a**, miR-190 expression in ERα-transfected MDA-MB-231 cells and control cells after E2 treatment was determined by RT-qPCR. **b**, The expression of SOX9 and ERα in cells as in (**a**) was determined by western blotting. **c**, The expression of ZEB1, SOX9, and ERα in ZEB1-transfected T47D and control cells. **d**, A model for the role of miR-190 in breast cancer anti-estrogen resistance. *P < 0.05

To further demonstrate that miR-190 mediates the regulation of SOX9 expression via ERα and ZEB1 in breast cancer cells, we transfected ERα plasmid into MDA-MB-231 cells in addition to E2 stimulation. The expression of miR-190 was increased, while that of SOX9 was decreased in ERα-overexpressing MDA-MB-231 cells in an estrogen-dependent manner, as determined by RT-qPCR (Fig. 7a) and western blotting (Fig. 7b). We next transfected ZEB1 plasmid into T47D cells and observed that the expression of SOX9 was increased in ZEB1-overexpressing T47D cells, accompanied with reduced expression of ERα and miR-190 (Fig. 7c and Fig. 6f). Collectively, our data support the conclusion that ERα and ZEB1 regulate the expression of SOX9 via regulating miR-190 expression in breast cancer cells.

## Discussion

Development of resistance to anti-estrogen therapy remains to be one of the major barriers to the successful treatment of patients with breast cancer. Our work suggested an important role of miR-190 in anti-estrogen resistance in breast cancer. First, we demonstrated that miR-190 inhibits Wnt/β-catenin signaling and increases the anti-estrogen sensitivity of breast cancer cells both in vitro and in vivo. Second, SOX9 was found to be a direct target of miR-190. Third, ERα and ZEB1 were shown to competitively bind to the miR-190 promoter and regulate its expression. Therefore, our results revealed a novel mechanism of constitutive Wnt/β-catenin signaling activation in anti-estrogen resistance in breast cancer and demonstrated that miR-190 functions as a tumor-suppressive miRNA in breast cancer.

miRNAs are small, non-protein coding RNAs first identified over a decade ago, and their dysregulation has been implicated in cancer development, progression, and drug resistance. Numerous miRNAs have been shown to be involved in acquired resistance to anti-estrogen therapies through regulating ERα and its interactors, growth factor receptor signaling, cell cycle regulators, and EMT [22]. miR-190 has been identified to be a tumor suppressor in several types of cancer, including neuroblastoma, prostate cancer, hepatocellular carcinoma, and breast cancer [10, 14, 23, 24]. Our previous study also indicated that miR-190 suppresses breast cancer metastasis and EMT phenotype through regulating TGF-β signaling [13]. Here, we observed that miR-190 increases the anti-estrogen sensitivity of breast cancer cells both in vitro and in vivo. CSCs possess the capacity for self-renewal and have the ability to drive the continued expansion of a population of malignant cells with invasive and metastatic propensity [25]. A growing body of evidence indicates that endocrine therapy-resistant breast cancer cells possess CSC characteristics [26]. Consistent with this, we showed that miR-190-overexpressing ER^−^ breast cancer cells have a decreased percentage of CD44^high^/CD24^low^ CSC population, and therefore, they will be more sensitive to tamoxifen.

SOX9, one of the members of the SOX family of transcriptional factors, plays a critical role in regulating developmental processes, including sex determination, chondrogenesis, neurogenesis, and neural crest development [27–30]. Many studies have demonstrated that SOX9 plays active roles during cancer tumorigenesis and progression in various types of cancer, including breast cancer [31]. Gene expression profiling has identified SOX9 as one of the signature genes that defines the basal-like subtype of breast cancer [32]. Furthermore, co-expression of SOX9 and Slug promotes the tumorigenic and metastasis-seeding capacities of breast cancer cells and is associated with unfavorable survival [33]. Consistently, depletion of SOX9 suppresses the tumor-initiating and metastatic abilities of breast cancer cells [31]. Recently, SOX9 was reported to be upregulated in tamoxifen-resistant breast cancer cells and drive breast cancer endocrine resistance [34, 35]. In our study, we identified SOX9 as a direct target of miR-190. More importantly, re-expression of SOX9 could reverse miR-190-induced increase in anti-estrogen sensitivity. These results indicated that SOX9 dysfunction contributes to the biological functions of miR-190 in breast cancer, especially anti-estrogen sensitivity.

The Wnt/β-catenin signaling pathway plays a critical role in adult-tissue homeostasis, stem cell self-renewal, and somatic cell reprogramming [36, 37]. Furthermore, this pathway regulates various processes that are important for cancer progression, including tumor initiation, tumor growth, cell senescence, cell death, differentiation, and metastasis, and the agents which can alter Wnt/β-catenin signaling have been used for clinical trials in preclinical models [38]. Emerging evidences have shown a close interaction between Wnt/β-catenin and ERα signaling [39, 40]. β-catenin has been shown to be associated with redistribution of ERα [41]. In addition, activation of Wnt/β-catenin signaling has been shown to be responsible for maintaining the stem cell-like characteristics of breast cancer cells, resulting in resistance to drugs, including tamoxifen [42, 43]. SOX9 has been identified to drive Wnt/β-catenin signaling pathway activation and cancer progression [19, 20, 44]. In line with these findings, we demonstrated that overexpression of miR-190 prevents the nuclear translocation of β-catenin and inhibits the activity of Wnt/β-catenin signaling. More importantly, re-expression of SOX9 could reverse miR-190-induced suppression of Wnt/β-catenin signaling. These results indicated that miR-190 inhibits the activity of Wnt/β-catenin signaling by targeting SOX9. ZEB1, a crucial member of the zinc finger homeodomain transcription factor family, is overexpressed in breast cancer cells and promotes breast tumorigenesis and cancer progression [45]. Recently, ZEB1 has been reported to repress ERα expression and attenuate cell growth inhibition by anti-estrogens [46]. Interestingly, we found a half ERE and two E-box binding sites on the miR-190 promoter. Furthermore, ERα and ZEB1 were found to competitively bind to the miR-190 promoter and regulate its expression. Taken together, our results showed that overexpression of ZEB1 could transcriptionally suppress the expression of ERα and miR-190 and led to SOX9 elevation and activation of Wnt/β-catenin signaling, resulting in anti-estrogen resistance therapies in breast cancer.

## Conclusions

In summary, we demonstrated that miR-190 inhibits Wnt/β-catenin signaling and increases anti-estrogen sensitivity by targeting SOX9. Furthermore, miR-190 is a transcriptional target of both ZEB1 and ERα. ERα and ZEB1 were found to competitively bind to the miR-190 promoter and regulate its expression. Based on the findings from this study and others, we propose a model that highlights the role of miR-190 in regulating Wnt/β-catenin signaling during breast cancer anti-estrogen resistance (Fig. 7d). The uncovering of the ZEB1/ERα-miR-190-SOX9 axis provides a better understanding of the Wnt/β-catenin network complexity. miR-190 may serve as a new option to target Wnt/β-catenin signaling for breast cancer intervention.

## Additional file

Additional file 1: **Supplementary Materials and Methods. Figure S1.** miR-190 increases tamoxifen sensitivity of breast cancer cells in vitro. **Figure S2.** Knockdown of SOX9 eliminates the effect of miR-190 depletion on tamoxifen sensitivity and stemness. **Table S1.** Primers used for RT-qPCR. (DOCX 1243 kb)

## Abbreviations

ChIP: chromatin immunoprecipitation
CSC: cancer stem cell
ERα: estrogen receptor-α
RT-qPCR: reverse transcription quantitative polymerase chain reaction
siRNA: small interfering RNA
SOX9: sry-related high motility group box 9
TGF-β: transforming growth factor-β
UTR: untranslated region
ZEB1: zinc-finger E-box binding homeobox 1
