# Supplementary material for: miR-190 enhances endocrine therapy sensitivity by regulating SOX9 expression in breast cancer
Source: J Exp Clin Cancer Res. 2019 Jan 18;38:22. doi: 10.1186/s13046-019-1039-9 (PMC6339391; doi:10.1186/s13046-019-1039-9)
Supplement: Supplementary file 1 — Supplementary Materials and Methods. Figure S1. miR-190 increases tamoxifen sensitivity of breast cancer cells in vitro. Figure S2. Knockdown of SOX9 eliminates the effect of miR-190 depletion on tamoxifen sensitivity and stemness. Table S1. Primers used for RT-qPCR. (DOCX 1243 kb) [file 13046_2019_1039_MOESM1_ESM.docx]

**Supplemental data**

**Supplementary Materials and Methods**

**Antibodies, reagents, plasmids, miRNA, and small interfering RNA (siRNA)**

Antibodies against SOX9, ZEB1 (Abcam, Cambridge, MA, USA), Ki-67, β-catenin, c-myc, CD44, ERα (Santa Cruz Biotechnology, Santa Cruz, CA, USA), TCF4, Cyclin D1, Nanog, Oct4, SOX2, and β-actin (Cell Signaling Technology, Beverly, MA, USA) were used. The ORF of human SOX9, ZEB1 and ERα generated from MDA-MB-231 and MCF7 cells, the resultant PCR product of which was connected together with pcDNA3.1 tagged HA. The SOX9 3ʹ-UTR containing miR-190 binding sites were amplified and cloned into psiCHECK2 vector (Promega, Madison, WI, USA) to generate Luc-SOX9. Site-directed mutagenesis was performed using the Site-Directed Mutagenesis Kit (TransGene, Beijing, China) to generate the SOX9 3ʹ-UTRmut reporter vector (SOX9M). The miR-190 promoter region (–300 to +1) and the E-box or/and ERE mutated fragments were cloned into pGL3-Basic vector (Promega; miR-190pW and miR-190pM1/2/3). All constructs were confirmed by sequencing. The miR-190 mimic, miR-190 inhibitor, or the appropriate scrambled controls were purchased from RiboBio (Shanghai, China). The ERα and ZEB1 gene-specific siRNAs, and non-specific control siRNA were also purchased from RiboBio.

**Cell culture**

MDA-MB-468, MDA-MB-453 and MDA-MB-435 cells were maintained in DMEM/F12 medium supplemented with 10% fetal bovine serum (FBS, Life Technologies). MDA-MB-231 cells were cultured in Leibovitz’s L-15 medium (Life Technologies) supplemented with 10% FBS without CO_2_ at 37°C. T47D and MCF7 cells were cultured in DMEM (Life Technologies) supplemented with 10% FBS.

**Cell proliferation assays**

MTT, plate colony formation and EdU assays were used to evaluate cell proliferation ability. For MTT assay, 24 h after transfection, cells were seeded into 96-well plates at a density of 5 × 10^3^ cells/per well. After incubation for the indicated time, cells were incubated with 10 μL MTT (0.5 mg/mL; Sigma-Aldrich) at 37°C for 4 h. The medium was then removed, and precipitated Formosan was dissolved in 150 μL DMSO. The absorbance at 570 nm was detected using a micro-plate auto-reader (Bio-Rad, Richmond, CA, USA). For plate colony formation assay, 24 h after transfection, cells were seeded in 6-well plates at a density of 500 cells/per well. After about 3 weeks, the colonies obtained were washed with phosphate buffered saline (PBS) and fixed with 10% formalin for 15 min at room temperature and then washed with PBS, followed by staining with hematoxylin. The number of colonies were counted and compared with control. The EdU assay were detected by the Cell-Light EdU Apollo488 In Vitro Imaging kit according to the manufacturer’s protocol (Ribobio). EdU-positive cells were calculated as (EdU add-in cells/Hoechst stained cells) × 100% under a fluorescence microscope.

**Supplemental Figures and Tables**


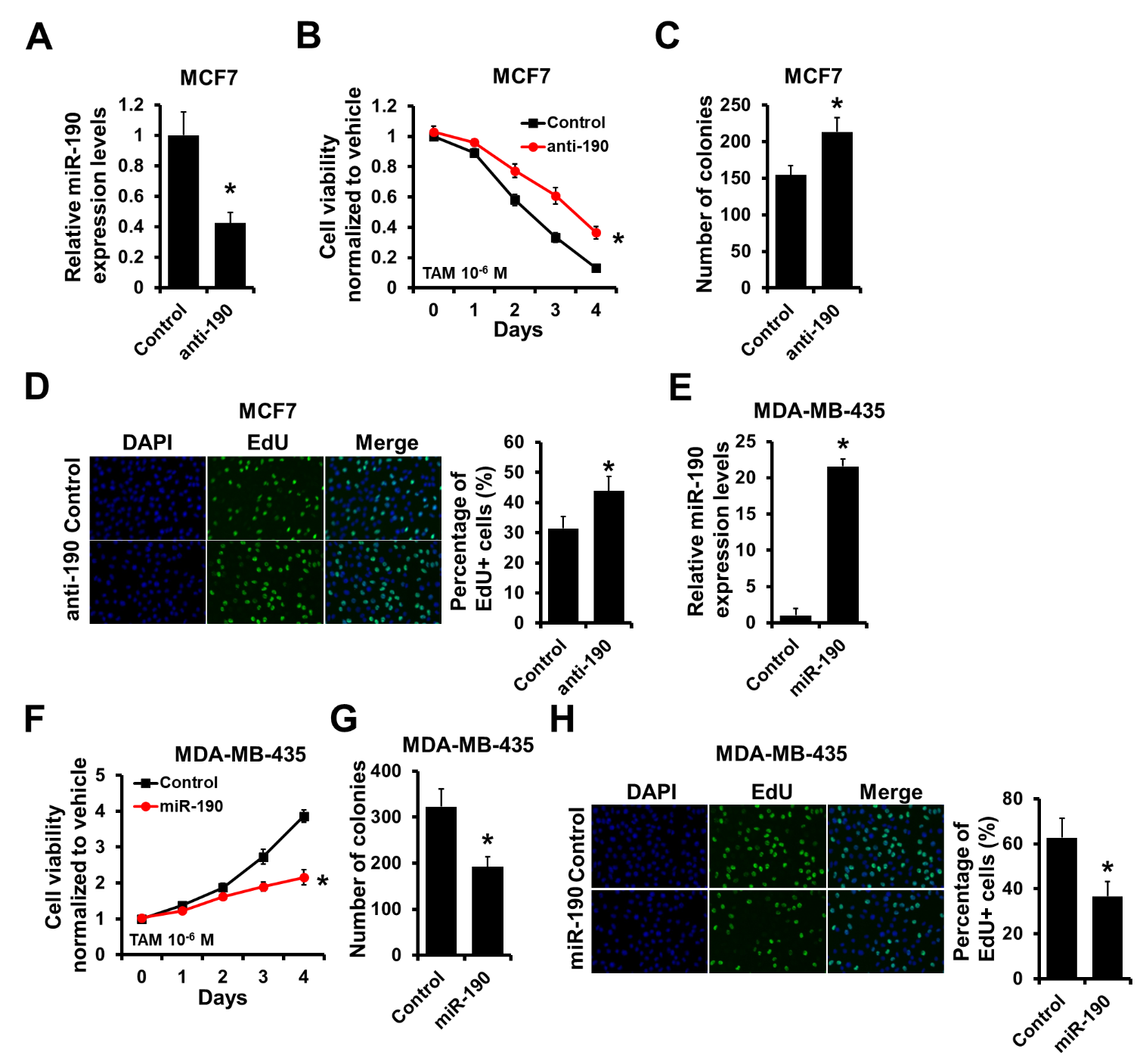


**Figure S1. miR-190 increases tamoxifen sensitivity of breast cancer cells *in vitro*.** A, The miR-190 expression in MCF7 cells transfected with miR-190 inhibitor as determined by RT-qPCR. **B - D,** Cell growth inhibition was determined by MTT (B), colony formation (C) and EdU (D) in MCF7 cells transfected with miR-190 inhibitor, as well as in control cells after treatment with tamoxifen. **E**, The miR-190 expression in MDA-MB-435 cells transfected with miR-190 mimics as determined by RT-qPCR. **F - H**, Cell growth inhibition was determined by MTT (F), colony formation (G), and EdU (H) in MDA-MB-435 cells transfected with miR-190 mimics, as well as in control cells after treatment with tamoxifen. **P* < 0.05.


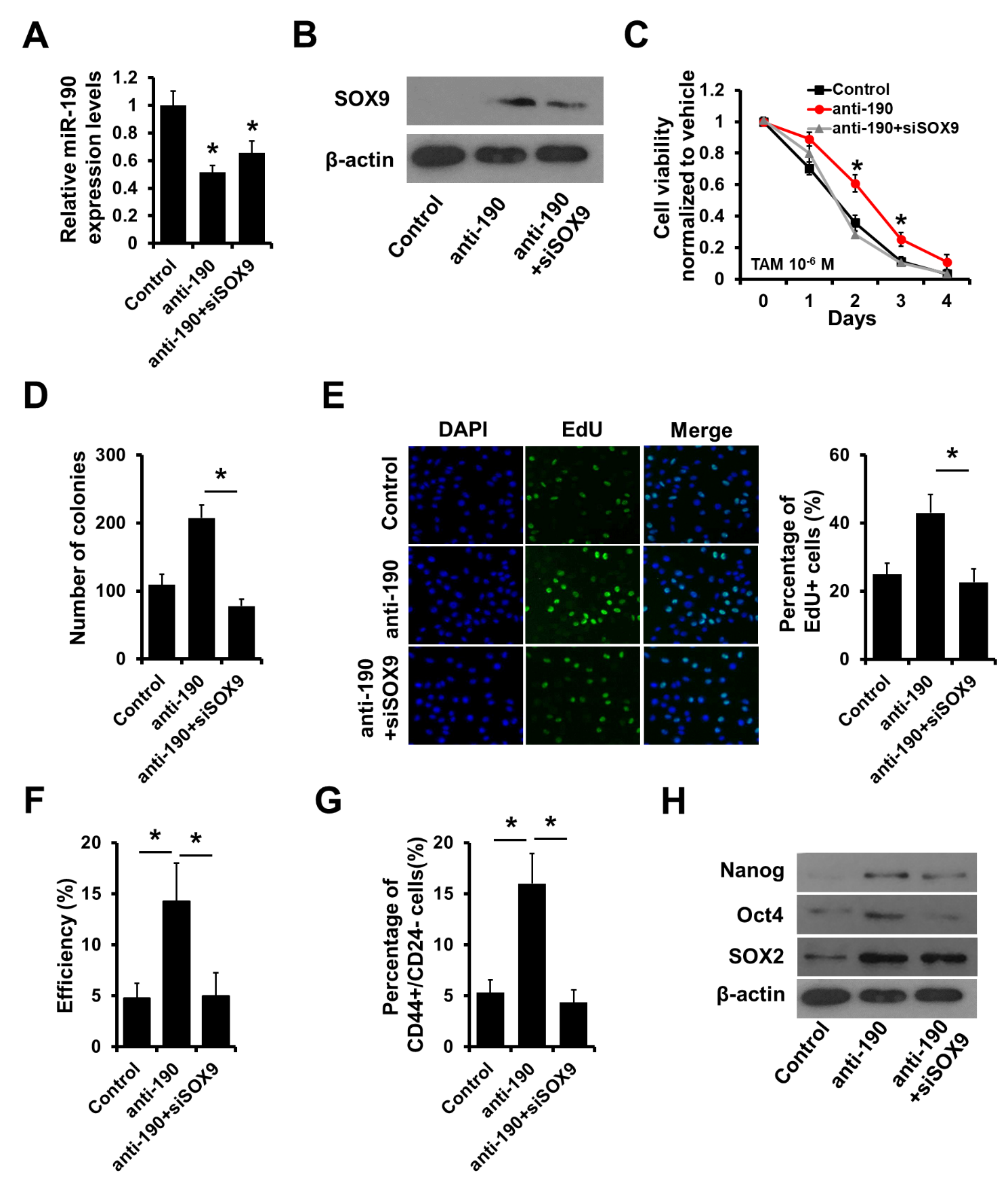


**Figure S2. Knockdown of SOX9 eliminates the effect of miR-190 depletion on tamoxifen sensitivity and stemness. A**, miR-190 expression in indicated T47D cells as determined by RT-qPCR. **B**, SOX9 expression in indicated T47D cells as determined by western blotting. **C~E**, Cell growth inhibition was determined by MTT (C), colony formation (D), and EdU (E) assays in T47D cells transfected with miR-190 inhibitor and siRNA targeting SOX9, as well as in control cells after treatment with tamoxifen. **F**, Mammosphere formation assay of cells as in (A). **G**, CD44^high^/CD24^low^ CSC population analysis of cells as in (A). **H**, The expression of CSC markers in cells as in (A) was determined by western blotting. **P* < 0.05.

**Table S1. Primers used for RT-qPCR.**

| Genes | Forward (5’- to 3’-) | Reverse (5’- to 3’-) |
| --- | --- | --- |
| ACTB | AGGCCAACCGCGAGAAGATGACC | GAAGTCCAGGGCGACGTAGCAC |
| SOX9 | AGTACCCGCACTTGCACAAC | CGTTCTTCACCGACTTCCTC |
| TCF4 | CCACCCATTTCTTTGCTGAAC | CCCTGACTCTTAACACCAACTC |
| CCND1 | GGGTTGTGCTACAGATGATAGAG | AGACGCCTCCTTTGTGTTAAT |
| CD44 | GCAGGTATGGGTTCATAGAAGG | GGTGTTGGATGTGAGGATGT |
| c-MYC | TGAGGAGGAACAAGAAGATG | ATCCAGACTCTGACCTTTT |
